# Developmental scRNAseq Trajectories in Gene- and Cell-State Space—The Flatworm Example

**DOI:** 10.3390/genes11101214

**Published:** 2020-10-16

**Authors:** Maria Schmidt, Henry Loeffler-Wirth, Hans Binder

**Affiliations:** IZBI, Interdisciplinary Centre for Bioinformatics, Universität Leipzig, Härtelstr. 16–18, 04107 Leipzig, Germany; wirth@izbi.uni-leipzig.de (H.L.-W.); binder@izbi.uni-leipzig.de (H.B.)

**Keywords:** pseudotime trajectories, transcriptomic landscapes, differentiation of tissues, planarian, machine learning, self-organizing maps, single cell RNA sequencing

## Abstract

Single-cell RNA sequencing has become a standard technique to characterize tissue development. Hereby, cross-sectional snapshots of the diversity of cell transcriptomes were transformed into (pseudo-) longitudinal trajectories of cell differentiation using computational methods, which are based on similarity measures distinguishing cell phenotypes. Cell development is driven by alterations of transcriptional programs e.g., by differentiation from stem cells into various tissues or by adapting to micro-environmental requirements. We here complement developmental trajectories in cell-state space by trajectories in gene-state space to more clearly address this latter aspect. Such trajectories can be generated using self-organizing maps machine learning. The method transforms multidimensional gene expression patterns into two dimensional data landscapes, which resemble the metaphoric Waddington epigenetic landscape. Trajectories in this landscape visualize transcriptional programs passed by cells along their developmental paths from stem cells to differentiated tissues. In addition, we generated developmental “vector fields” using RNA-velocities to forecast changes of RNA abundance in the expression landscapes. We applied the method to tissue development of planarian as an illustrative example. Gene-state space trajectories complement our data portrayal approach by (pseudo-)temporal information about changing transcriptional programs of the cells. Future applications can be seen in the fields of tissue and cell differentiation, ageing and tumor progression and also, using other data types such as genome, methylome, and also clinical and epidemiological phenotype data.

## 1. Introduction

Genome-wide single cell transcriptomics experiments provide snapshot data, which resolves the molecular heterogeneity of cell cultures and tissues with single cell resolution under static conditions [1,2]. These measurements are cross sectional and lack explicit time-dependent, longitudinal information about the developmental dynamics of each individual cell. Given that each cell can be measured only once, one needs models and computational methods to deduce developmental trajectories on cellular level and changes in underlying molecular programs from these static snapshot data. Such methods were developed in order to quantify transcriptional dynamics such as cell differentiation or cancer progression by using the concept of pseudotime (pt) [3,4,5,6]. The pt model assumes that single cell transcriptomes of different cells can be understood as a series of microscopic states of cellular development that exist in parallel at the same (real) time in the cell culture or tissue under study. Moreover, the model assumes that temporal development smoothly and continuously changes transcriptional states in small and densely distributed steps so that similarity of transcriptional characteristics can serve as a proxy of time. Here the pt represents the similarity measure used. It scales development using values between zero and unity for the start and end points, respectively. Pt methods typically project the high-dimensional molecular data on to a space of reduced dimensions by (non-)linear transformations. In reduced dimensional space the cells were then aligned along a trajectory scaled in units of pt where a large variety of projection algorithms can be applied (see, e.g., [7,8,9]). A recent benchmarking study identified more than 70 pt-trajectory interference methods. About 45 of them were explicitly evaluated using criteria such as cellular ordering, topology, scalability, and usability [10]. Each method has its own characteristics in terms of the underlying algorithm, produced outputs, and regarding the topology of the pt trajectory. Methods make either use of pre-defined, fixed path topologies such as linear [3,11], cyclic, or branched [4,12,13] or they infer the topology from the data, e.g., as connected or disconnected graphs [12,14,15]. Most methods aim at inferring continuous cell state manifolds. To achieve this they transform single-cell data to graphs representing the individual cells as nodes, which are then connected by edges that reflect pairwise gene expression similarities. Such graph-based analyses are useful because they convert a set of isolated measurements of single-cell transcriptomes into a connected structure, which can then be analyzed using a rich set of mathematical methods for construction and visualization of the state space manifold and for (pseudo-)temporal analysis (see [16] and references cited therein). Method’s performance depends on the trajectory type, dimensions of the data, and prior information where however often little is known about the expected trajectory. Notably, also different kinds of network studies aimed at inferring trajectories as directed graphs, e.g., in the context of metabolic flux analyses ([17] and references cited therein).

Hence, pt trajectories refer to ordered series of cell states. Alterations of activities of selected genes or gene sets along these trajectories then provide pt profiles of gene expression, which represent x-y plots depicting the expression levels as a function of pt [18]. They characterize (pseudo-)temporal changes of cellular programs upon development and can proceed, e.g., in a switch-like or in a more continuous fashion, or they can upregulate in intermediate, transient states [19]. Accordingly, molecular developmental characteristics can be split into two orthogonal views, namely focusing either onto the cells as the functional unit or onto molecular programs as changes of function independent of the associated cell state(s). Both aspects are closely related but not identical because development into different cell types can be driven by the same or by different molecular processes and, vice versa, different programs can associate with one or multiple cell types. For example, co-evolution of tumor cells and their microenvironment involves different cell types and states, which are expected to show co-regulation in gene-state space and potentially could support inference of the development of molecular communication networks between different cell types (see [20,21] and references cited therein). Trajectories in cell-state space are addressed by the numerous pt-approaches mentioned above while elaborated methods and applications for visualization and analytics of developmental paths in gene state do not exist to the best of our knowledge.

We previously developed the expression portrayal method that visualizes multidimensional, whole genome expression landscapes in terms of two-dimensional images making use of self-organizing map (SOM) machine learning [22,23]. Such landscapes are typically characterized by clusters of genes, so-called spot modules, concertedly overexpressed under certain condition such as cell types or developmental stages. The self-organization properties of the method arranges the spot-modules of similar expression patterns closer to each other than more dissimilar ones [22]. Hence, SOM machine learning arranges spot-clusters of co-regulated genes according to mutual similarities of their expression profiles in an ordered fashion resembling paths in gene space. In other words, SOM accomplishes the task of sorting features in gene-state space in analogy to the task of sorting cells according to mutual similarities of their transcriptomes along pseudotime trajectories in cell-state space.

We have previously shown that the arrangement of spots indeed reflects dynamics of different processes. For example, melanoma progression from naevi to metastatic tumors has been analyzed by combining SOM portrayal and pt-scaling of bulk melanoma transcriptomes [24] and single-cell data of melanoma cell cultures [19,25]. Another study addressed organoid development of liver buds using a similar approach [26]. The ability to deduce dynamic information in SOM gene-state space from cross-sectional data was supported by (real) time-resolved SOM-analyses of the longitudinal transcriptomic changes along the yeast cycle [27], of dynamic changes of urine proteomics during space flight simulations of human individuals [28] and, earlier, equilibration of cellular states after external perturbations into stable attractor states [29,30]. In these applications SOM provided trajectories in the molecular landscapes. The studies so far associate with relatively simple linear, circular, bifurcation-like, and converging processes. Furthermore, the trajectories in feature-space were deduced more on a qualitative level without underpinning them with appropriate computational methods. Finally and most importantly, single cell “omic” technologies strongly evolve and now became standard in many applications beyond the flatworm example used here. Presently, many ten-thousands of cells are sequenced in parallel, quality of transcriptomic data is improved, technology is refined, e.g., in direction of spatially resolved transcriptomics, and extended by single-cell proteomics, genetics, and chromatin accessibility methods.

In this publication, we aim at further developing the idea of constructing developmental trajectories in SOM-feature state space from cross-sectional data in order to supplement pt trajectories in cell-state space with such trajectories describing developing molecular programs. Our efforts should be understood as a first step in this direction as a proof of principle approach. It extends previous work by explicitly extracting “similarity paths” in the expression landscape provided by the SOM method. In other words, we supplement our portrayal of static molecular landscapes by (pseudo-)dynamical views in analogy to pt approaches in cell-state space.

Making use of topological features of the expression landscape we also process so-called RNA-velocities as an independent approach. RNA-velocity analysis directly “forecasts” the transcriptional state of a cell based on the relation between spliced and unspliced mRNA [31,32]. We here apply this concept to gene-state space to derive local gradients of transcriptional activity pointing toward attractors of gene activity along RNA-velocity paths. Hence, as the second novelty we project RNA-velocity information into expression portraits.

We selected “flatworm” single cell RNAseq data as an illustrative example. Planarian transcriptomics was well studied as a first application of scRNAomics to developmental dynamics in a whole complex animal [29]. This data enabled to reconstruct multibranched lineage relationships of cell differentiation from stem cells into different tissue types and to identify gene sets, which program the lineage tree. The animal overall consists of several tissue types, so that multibranching of developmental trajectories is sufficiently diverse.

**Figure 1:**
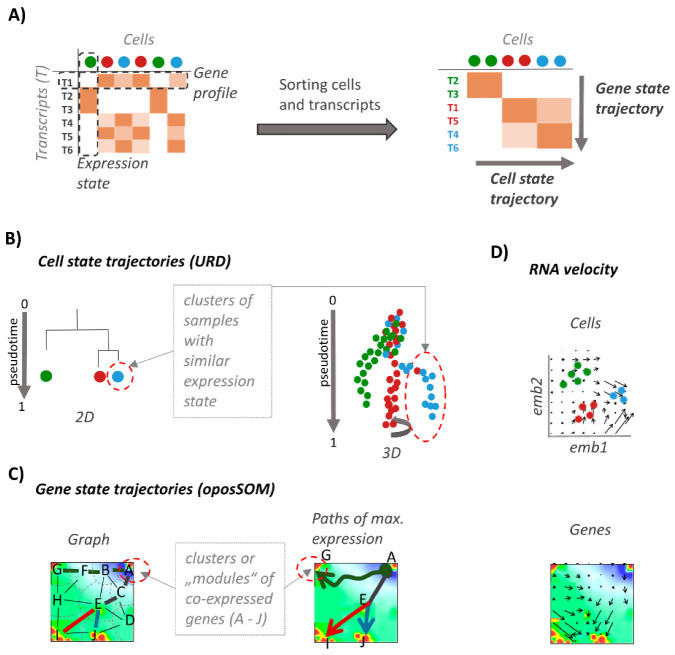
Analysis of developmental paths (schematic overview). (**A**) Single-cell transcriptome data are provided as a matrix of cells (columns) versus transcripts (rows). Similarity sorting along genes and cells is applied to obtain gene- and cell-state trajectories, respectively. (**B**) Multibranched cell-state trajectories order cells along a similarity measure called pseudotime (pt). They are visualized either as hierarchical tree or as three-dimensional manifold using, e.g., “URD”-plots [29]. They reflect transcriptional changes upon development and differentiation of cells. (**C**) The gene-state trajectories were generated in self-organizing map (SOM)-space making use of a network formed between clusters of co-regulated genes marked with letters in the figure. Trajectories follow paths of maximum expression linking source (stem cells) and sink (differentiated tissues) nodes along the edges of the net. (**D**) RNA velocity analysis delivers vector fields in cell and gene phase space. Each vector points in direction of increasing transcript abundance either between cell states or between gene states. In fact, RNA velocity forecasts the “future” transcriptional state of each gene. The RNA-velocity vector of the cells is formed by the RNA-velocities of all genes as components while the RNA-velocity vector in gene space is composed by the RNA-velocities of all genes in the respective local gene cluster.

Our publication is organized as follows (as shown in Figure 1): first, we introduce both cell- and gene-state centric views on development of Planarian tissues. Second, we characterize the SOM-expression landscape and extract differentiation paths into selected tissue types. For comparison with cell-state space we made use of the URD-multibranched diffusion pt method. Finally, we mapped RNA-velocities into SOM to derive vector fields of changes of RNA abundance. Overall, the SOM-based gene expression analysis of developmental trajectories complements cell-trajectories by more clearly disentangling involved molecular programs.

## 2. Materials and Methods

### 2.1. scRNA Seq Data of Planarian Development

Single-cell RNA Seq data of a whole planarian Schmidtea mediterranea were taken from the publication of Plass et al. [29] as read-counts (in units of RPKM) of more than 28,000 transcripts per cell. Originally these data were measured by highly parallel droplet-based single-cell transcriptomics, Drop-seq [33], to study different cell types and progenitor stages present in adult planarians. In total 21,612 cells were captured in 11 independent experiments. The data set corresponds to five wild-type samples (10,866 cells), two RNA interference (RNAi) samples (3314 cells), a high-DNA content G_2_/M population corresponding to cycling planarian stem cells (typically defined as x-ray–sensitive “X1 cells”; 981 cells), and three wild-type regeneration samples (6451 cells) [34,35]. As in the original publication [29], we analyzed all single-cell data sets together. Cell types were assigned to the previously annotated 51 cell clusters. Cell type identities of neoblast, neural, epidermal, secretory, muscle, gut, and protonephridia cells were further elucidated by examining known marker genes based on previous knowledge [29]. We applied the same color scheme as in the figures shown in [29] throughout this publication for direct comparison.

### 2.2. Single Cell SOM Portrayal

For transcript-related analysis we applied the “Single-Cell R Analysis Toolkit” available as R-package “scrat” [22,23,27]. In brief, it works as follows: Transcript-level expression data were log-transformed and centralized into log-fold changes with respect to the ensemble average of each transcript, log FC (cell) = log expression (cell)—mean_log expression (all cells). Then, SOM neuronal network machine learning translates the high-dimensional expression data of N = 28,065 gene transcripts into K = 3600 metagene expression data per cell. Implementation of SOM and optimal parameter settings were addressed previously [27]. Each metagene represents a “micro”-cluster of co-expressed genes showing mutually similar expression profiles across the samples. Metagenes were arranged in a 60 × 60 two-dimensional grid coordinate system and colored according to their expression level in each sample thus generating an image of the transcriptome of each single cell. The resolution of 60 × 60 metagenes was chosen to optimally resolve expression modules as shown previously. Default coloring of metagenes (red to blue for maximum to minimum expression, respectively) scales with log-expression values [22,36]. Mean portraits of the transcriptome of a cell type were calculated by averaging metagene expression values over all single cell portraits of the respective type.

Metagenes of mutually correlated profiles cluster together forming “spot-like” red and blue areas of over- and under-expression in the portraits because of the self-organizing properties of the SOM. These “spot”-modules were detected using distance-metrics (D-map) clustering [37]. They extract groups of co-expressed genes showing high expression in specific cell types. However, the spots cover only part of the SOM and thus only part of the genes. For “space-filling” segmentation of the SOM we applied k-means clustering to all metagenes. This k-means clustering stratified the 3600 metagenes into 32 clusters where 11 of them can be assigned to the spots, 21 collect virtually low variant transcripts, and one cluster collects almost invariant genes (see below). The choice of the number of metagenes, clustering criteria, and methods for segmenting SOM were extensively studied and described previously [22,27]. In short, a number of several thousand metagenes are large enough to resolve transcriptional landscapes in an unsupervised fashion in order to identify dozens of different states with sufficient resolution. Larger numbers of metagenes have virtually no effect on the results. The number of spots represents an intrinsic property of the data. It is obtained in an unsupervised way by searching for modules of co-expressed genes by applying different algorithms based on overexpression or D-map criteria. Typically their number is roughly comparable with the number of transcriptomic states as has been shown in previous applications [25,36,38]. It is expected to vary between ten and twenty in the flatworm example. For low-variant modules a number exceeding the number of spot modules by a factor of two is appropriate to segment the map with sufficient resolution. A number too small (e.g., five or less) will provide a too coarse resolution of the paths while higher numbers (e.g., more than thirty) do not change the results and, moreover, typically produce “empty” clusters without functional information.

The spot-patterns obtained represents a characteristic fingerprint of each particular sample. This “logFC” scale is used as default color scale of the portraits (see also [22]). It enables to identify well-separated areas of overexpression (red color) as described above. In addition, we applied a so-called “loglogFC” scale for alternative coloring. It applies the signed logarithm of the absolute value of logFC, loglogFC = sign log(abs(logFC)), where “sign” equals the sign of logFC. First of all, the “loglogFC” scale identifies a “coast-line” which separates areas of overexpression (FC > 1) in red from areas of underexpression (FC < 1) in blue. LoglogFC portraits overall better resolve subtle changes of the expression landscape in low expression areas.

### 2.3. Cell Clustering, Cell State Trajectories, and Pseudotime

For an overview, we visualized similarities between transcriptomes of planarian single cells using t-distributed stochastic neighbor embedding (tSNE) algorithm [39]. Pseudotime (pt) analysis was performed by means of simulated diffusion paths through the cells in similarity space as implemented in URD [40]. It distributes the scRNAseq transcriptomes along a branched tree-structure reflecting different trajectories of cell development. We assigned the root of differentiation to planarian stem cells. Then URD calculates the trees by constructing a k-nearest-neighbor graph and assigns a pseudotime (pt) by simulating a diffusion-like process from the root to the tip of the graph. The pt scales similarities between the cell transcriptomes using a value between pt = 0 at the start to pt = 1 at the tip of largest distance. Overall application of URD delivers a branched trajectory from one root to several tips.

### 2.4. Gene State Trajectories in SOM Space

Gene-state trajectories were calculated by applying the maximum flow algorithm [41] to transcriptomic data in metagene space as provided by SOM. To generate these trajectories, we first segmented metagene space into space-filling k-means clusters as described above. Then they were transformed into a network with edges linking all neighboring clusters. The weights (called capacities) of the edges were given by the cumulative mean loglog FC value of the two adjacent clusters linked by the edge. Next, we defined a source cluster (starting point) and the sink cluster (end point) of the differentiation process, e.g., between genes upregulated in undifferentiated stem cells and genes upregulated in differentiated tissues. The maximum flow-algorithm connects source and sink along the edges providing the maximum cumulative weights. This path then defines the gene-state trajectory ensuring maximum cumulative expression between source and sink. Alternatively we made use of different portraits between differentiated tissue and stem cells to generate gradient trajectories along topographic least cost paths from source to sink spots using the R-program topoDistance [42].

### 2.5. RNA-Velocity Dynamics

The so-called RNA-velocity is the time derivative of gene expression. It is positive for increasing and negative for decaying transcript abundance. It can be evaluated from the relative amounts of unspliced and spliced mRNAs of each gene taken from RNAseq data, their ratio under steady state conditions and assuming a constant splicing rate of all genes [31]. The mRNA-velocities of all genes form a multidimensional vector pointing in direction of the overall increment of mRNA of the cell and thus predicting its future expression state. In metagene space as provided by SOM the mRNA velocity of a metagene is given by the multidimensional vector with the mRNA-velocities of all single genes included in the metagene cluster as components. It points toward the future state of the metagene in the expression landscape, or, in other words, it forecasts the expression change of the metagene. Overall, the metagene-related mRNA-velocities thus provide vector fields pointing toward local attractors of maximum transcription (see below). The vector field obtained is then transformed into trajectories using RNA-velocity [31]. It summarizes the metagene-related RNA-vectors into “flow”-vectors toward the sinks (or attractors) of transcription (see below).

### 2.6. Function Analysis

We applied gene set analysis to the lists of genes located in each of the modules to discover their functional context using the planMine database [43]. planMine takes the Gene Ontology (GO) information for a transcript from the NCBI database for RefSeq proteins that show strong Blast homology with Planarian transcripts [43]. Gene set maps complement this analysis by visualizing the position of the genes of a set within the SOM grid. According to their degree of accumulation in or near the spot modules, one can deduce their potential functional context [22].

### 2.7. Methods Availability

Trajectory generation and visualization is implemented as R-package oposSOM.PT (https://github.com/mschmidt000/oposSOM.PT). It should be used together with the R-package oposSOM [19,20] (https://github.com/hloefflerwirth/oposSOM) in order to generate trajectories upon running the program.

## 3. Results

### 3.1. Portrayal of Developing Single Cell Transcriptomes

**Figure 2:**
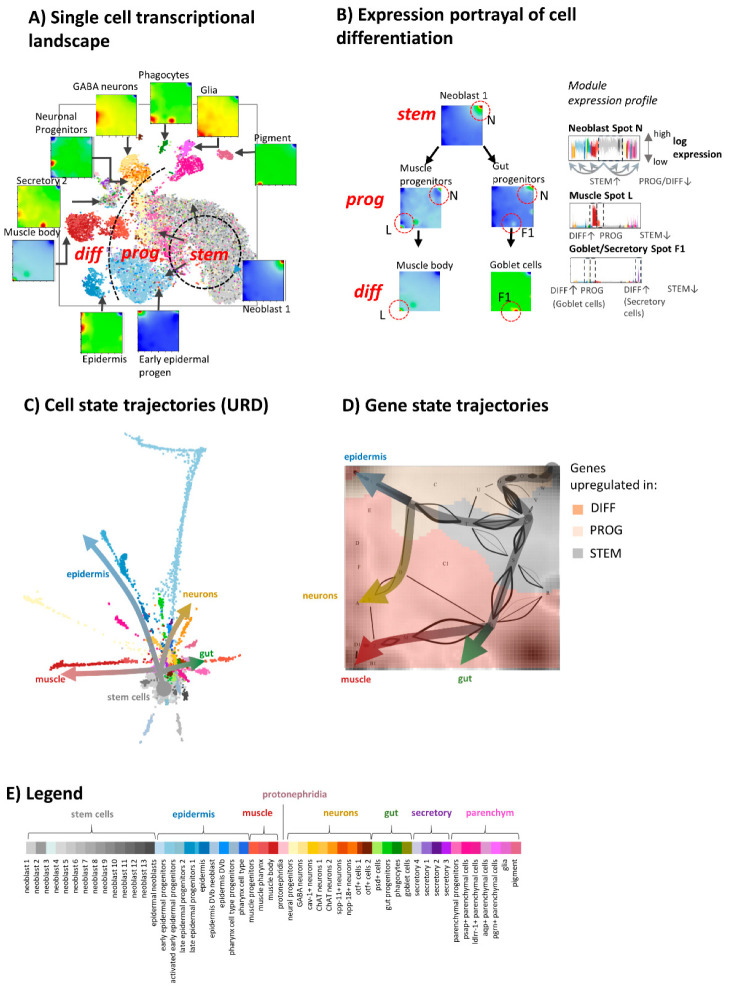
SOM portrayal of the developing planarian single cell transcriptome: (**A**) Single-cell resolved tSNE plot (cell types are color-coded as shown in the legend in part E). SOM expression portraits of selected cell types provide a glimpse on cell and gene transcriptional space. (**B**) Expression portraits of stem cells and of differentiating progenitor and differentiated muscle body and a goblet cells show overexpression spots at different positions as indicated by the dashed circles. The spot profile reveals specific up regulation of the spots in the respective tissue specific cells. (**C**) The URD [40] plot resolves multibranched single-cell developmental manifolds. Paths of selected tissue types are indicated by arrows. (**D**) Gene activation trajectories of cell differentiation from stem cells into four different tissues were calculated by means of maximum flow on SOM embedding. (**E**) Legend of colors used for cell-type assignment (see also [29]). The color code is used throughout the paper.

For the comprehensive cartography of cell states upon tissue development of planarian, we generated SOM transcriptomic portraits of all nearly 22,000 cells studied. Then, we summarized them into mean portraits of each of the 51 cell types defined previously [29] by averaging all single cell-portraits of each type. tSNE plots were used for an overview of transcriptional diversity of the SOM portraits (Figure 2A). Cells were grouped into 51 clusters in accordance with the classification and coloring scheme used previously [29] (see legend in Figure 2E). Our SOM-based results are in line with that of Plass et al. [29] showing one large cluster of neoblast stem-cells (grey color) which is surrounded by clusters of differentiated cells of different tissues (dark color tones) and clusters of progenitor cells (brighter colors) in between (Figure 2A). The “differentiation” axis points roughly from the stem cells in the right part toward differentiated cells in the left. The SOM expression portraits of the cell types are characterized by red and blue spots at specific positions. They refer to clusters of concertedly over- and under-expressed genes, respectively. For example, neoblasts show a red overexpression spot in the upper right corner of the map, while differentiated muscle and goblet cells show overexpression spots in the left and middle bottom area, respectively (Figure 2B). The respective progenitor cell express a virtually bimodal spot patterns consisting of a mixture of the spots observed in the undifferentiated and differentiated cells, respectively. One can understand differentiation as a series of activated cellular programs, which become evident as path in the expression landscape linking the stem cell spot with the spot observed in differentiated cells. Examples of such developmental trajectories of changing gene states were shown in Figure 2D for muscle, gut, epidermal, and neuronal cells obtained by means of the maximum flow algorithm (see Materials and Methods section). Changing cell states upon differentiation were visualized using URD-clustering. This method sorts cells according to mutual similarities along pseudotime trajectories, which are specific for different developmental paths (Figure 2C).

In summary, we here introduced two complementary views on developmental trajectories, namely as paths describing the sequence of cellular states passed between stem cells and differentiated tissue cells on one hand and as the sequence of major gene regulatory programs activated upon differentiation on the other hand. SOM-space visualizes the latter aspect. It enables studying functional aspects of development in gene space.

### 3.2. The Transcriptome Landscape of Planarian

**Figure 3:**
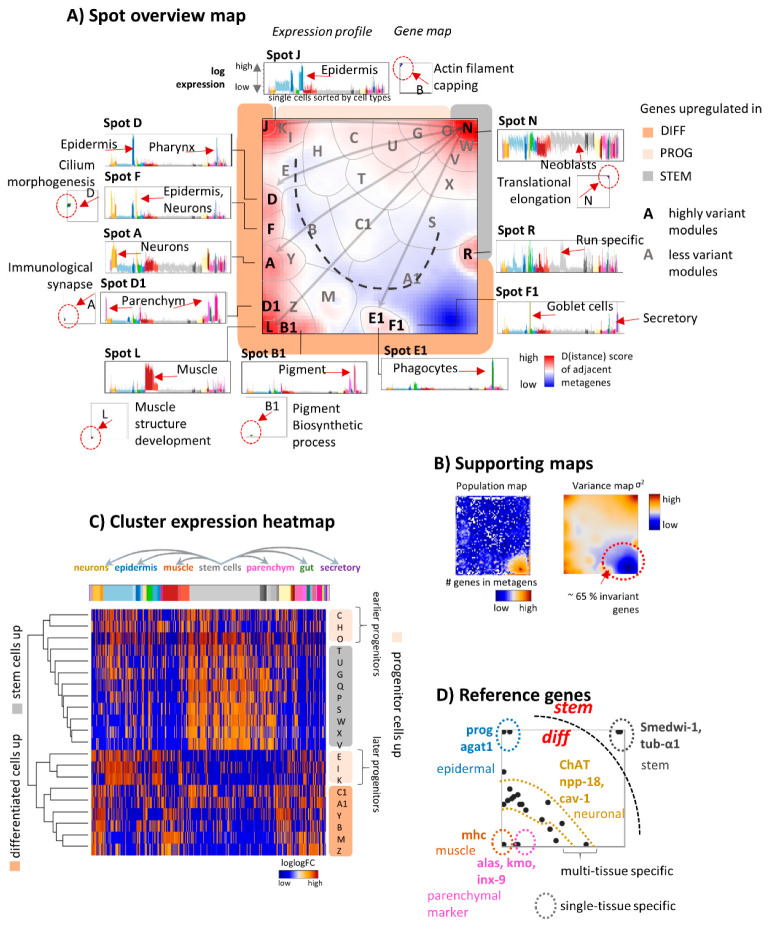
The single-cell transcriptomic landscape of the planarian Schmidtea mediterranea: (**A**) The module overexpression map provides an overview about modules of co-expressed and highly variant genes. They mainly arrange along the edges of the SOM and are labeled with bold capital letters A, D, F, J, L, N, R, B1, D1, E1, F1. The grey arrows illustrate selected developmental paths by linking the stemness spot N with the respective “tissue” spots. Expression profiles (gene set enrichment Z-score, GSZ-scale) and gene maps of selected functional gene sets underpin the functional context of these modules. They reveal specific up- and downregulation of the gene sets in different tissues (red arrows). The gene maps indicate the location of the genes by dots. Accumulation of genes in and near module areas are indicated by red arrows and dashed ellipses. (**B**) Population and variance maps visualize the number of genes in each metagene (log-scale) and the variance of metagene expression, respectively. About 65% of all genes studied show virtually invariant expression. They accumulate in the blue area in the right lower corner as indicated. (**C**) Expression profiles of the modules of low variant expression (labelled with non-bold letters in part A) are shown as heatmap. The arrows above the heatmap point in the direction of differentiation from stem cells into different tissues. The spot modules group roughly into three clusters as indicated in the right part of the heatmap and along the edges of the SOM in part A. (**D**) Location of key reference genes are shown by dots (see also Figure S2 and Table S1). They are mostly found in or near the spots which upregulate in the respective tissues.

Next, we further characterized the topology of SOM expression landscape and its functional context in detail. In total, we segmented the 3600 metagenes used for the SOM training into 32 expression modules by applying k-means clustering to the metagene’s expression profiles. The modules were labeled by capital letters “A–F1” (Figure 3A). A summary of the top expressed marker genes in each of the modules, enriched functional gene sets, and samples showing upregulation are provided in Table 1. The modules are divided into 11 highly variant expression clusters (A, D, F, J, L, N, R, B1, D1, E1, F1) and into 21 less variant clusters. The former ones form “spot-like” red areas along the periphery of the SOM which specifically upregulate in the different tissues (Figure 3C). The low variance clusters were found in the middle and right lower part of the map. About 65% of all genes are virtually invariant and accumulate in cluster A1 evident as blue area in the summary and variance maps (Figure 3B). Hence, the topology of the SOM splits into one developmental source spot-collecting genes overexpressed in stem cells, into a series of developmental sink spots-collecting genes upregulated in differentiated tissues and into an intermediate transition region in between. Further, SOM identified an area collecting virtually invariant genes and spot R of unknown origin. Presumably, it can be attributed to methodical effects systematically disturbing parts of the expression patterns.

The landscape is governed by the amplitudes and particular shapes of the gene expression profiles. They are shown around the map for selected modules together with gene maps indicating the positions of gene referring to specific Gene Ontology (GO) terms (Figure 3A). For example, genes of the GO-term “Muscle structure development” accumulate in and near the “muscle” module L upregulated in muscle tissue this way confirming its functional impact. The heatmap in Figure 3C visualizes the profiles of the less variant clusters. Overall, these profiles split into three major strata either upregulated in stem cells (marked with grey color), progenitor cells (light apricot), or differentiated cells (apricot), respectively (Figure 3A–C). The progenitor cell clusters can be further divided into “earlier progenitors” activated still in stem cells and into “later progenitor cells” not showing activation in stem cells. Marker genes of differentiation between the different planarian cell types (Table S1 and [44]) map into or near the modules upregulated in the respective cell types (Figure 3D). For example, the epidermal module J includes the epidermal gene markers prog-1 or agat-1 while the stemness module N contains the stem cell marker Smedwi-1 and tub-α1. Overexpression analysis of selected reference sets of co-regulated genes also well agreed with our modules. They further specify functional interpretation, e.g., in terms of activated cell cycle phases within the stem cell compartment (see Figure S1B–E).

Note that spots and underlying cellular programs are activated in different cell types (see the profiles in Figure 3A). For example, cell-cycle-related transcription is virtually active in all cell types however to a different extent (Figure S1D). These genes locate mostly in spots W and V near the stemness module N. Other common programs are more selective: genes related to morphogenesis accumulate in spot F upregulated in epidermal and muscle tissues. Such genes activated in several differentiated tissues overall accumulate in a stripe-like area near the lower left corner of the map including spots F, Y, and M (Figure 3A,D).

Gene set enrichment analysis using the database planMine for mining flatworm genomes and transcriptomes [43] provided detailed functional information associated with each cell type (Table 1). For example, the top enriched sets in the “muscle module” L are the GO-terms collagen fibril organization, sarcomere organization, and muscle tissue development while the “stem cell module” N enriches terms such as rRNA processing, ribosomal subunit biogenesis, and RNA processing, reflecting the high biogenetic and metabolic activity of stem cells. In summary, the diversity of the scRNAseq data decomposes into a set of one dozen clusters of co-regulated genes of defined functional context. Along the differentiation axes the cells split into neoblasts, early, and late progenitors and differentiated tissue cells according to their transcriptomic patterns.

### 3.3. Portrayal of Developmental Paths

**Figure 4:**
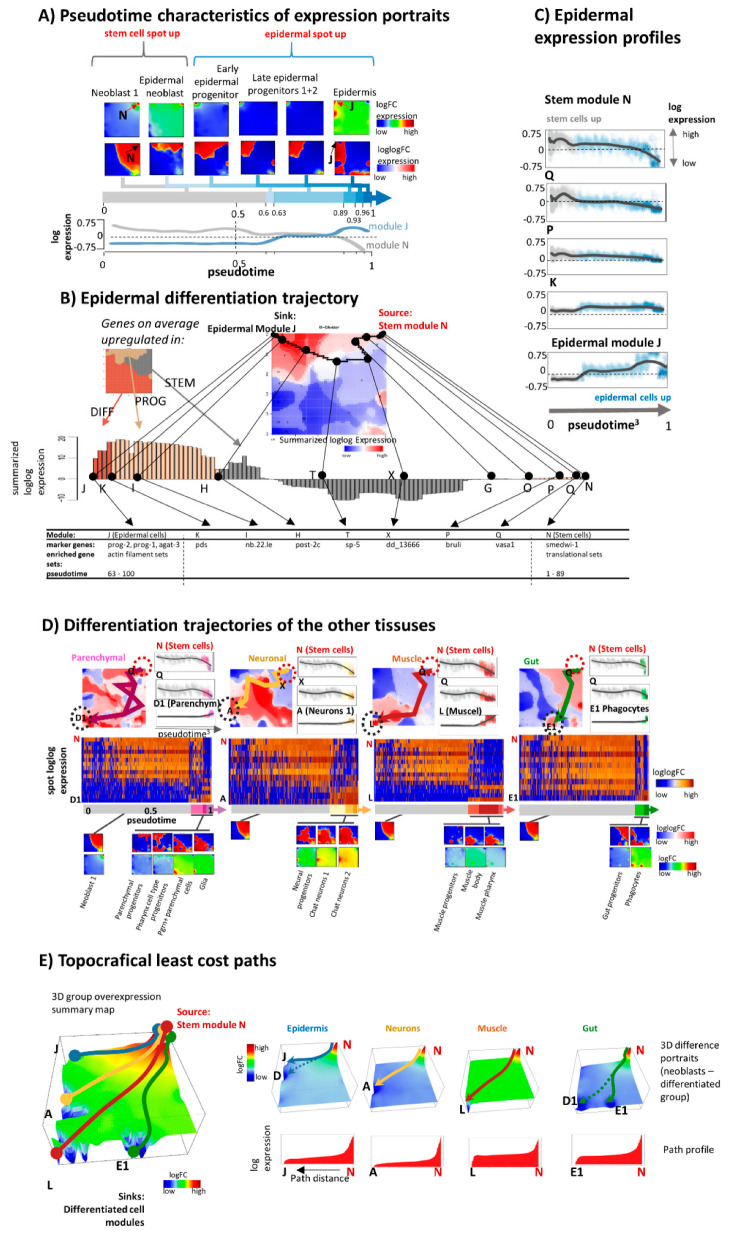
Gene expression trajectories of planarian tissue development. (**A**) Expression portraits (in log FC and loglog FC scales) of the epidermal lineage illustrate the change of module-patterns during differentiation. The “loglogFC” scale better resolves subtle changes between over- and under-expression in red and blue, respectively. Alterations of expression patterns become evident as “lava lamp”-like flow of the red spot from the right to the left. The expression of stemness and epidermal spots N and J change in an antagonistic fashion along the pseudotime. (**B**) The gene-state trajectory of epidermal development links the stemness spot with the “epidermal” spots in the expression landscape shown as cumulative loglogFC SOM). The expression profile along the trajectory assigns genes and functions along the path. (**C**) Selected expression profiles of spots along the developmental path of epidermal cells switch from negative to positive slopes between spots P and K. (**D**) Developmental characteristics of four different planarian tissues. The row above shows SOM-trajectories and pt-profiles. Below, the heatmaps of expression modules characterize expression changes of the respective tissues upon development as a function of pt. The respective expression portraits are shown below. (**E**) Gene state developmental trajectories obtained from the difference of SOM between stemness and differentiated tissue states using “topoDistance” analysis show differentiation paths as in part A–D. Largest expression changes were observed in the “final” slope describing differentiation of tissues from progenitor states.

In the next step, we aimed at characterizing gene-state expression dynamics during differentiation. For a simple first view, we ordered the expression portraits of the cells of epidermal differentiation along the linear, unbranched pseudotime path taken from Plass and colleagues [29] (Figure 4A). Hereby the stemness module is highly expressed in the portraits of stem cells, of epidermal neoblast, as well as in epidermal progenitors. Its amplitude however progressively decays in the portraits of early and late epidermal progenitors but then markedly decays in the portraits of differentiated epidermal cells. On the other hand, the expression of the epidermal module progressively increases in an antagonistic fashion. Notably, this switching affects about 1000 genes included in these two modules. We replot the default “logFC”-scaled portrait into double-logarithmic “loglogFC” scale in order to better visualize subtle expression changes upon differentiation. The red overexpression region “flows” along the upper border of the map in direction from the stemness “source” spot at the right toward the epidermis “sink” module at the left. A gene-state trajectory of epidermal differentiation was computed using the maximum flow algorithm [10] as described in the methods section. Briefly, the trajectory is obtained as directed graph linking the source and sink module via the path of maximum cumulative expression between the adjacent modules along the path (Figure 4B). As expected, the trajectory follows the right-to-left shift of the red overexpression region seen in the portraits upon differentiation. The expression of stemness genes decays along the trajectory and it increases for genes involved in epidermis differentiation (Figure 4C). Gene-state trajectories of differentiation, pt profiles, and associated expression portraits of parenchymal, neuronal, muscular, and gut tissues are shown in Figure 4D. Hereby, differentiation is described by trajectories starting in the common stemness mode and ending in the respective tissue-specific expression modules.

As an alternative method we applied a lowest cost path algorithm to the difference map between stemness and differentiated tissues (Figure 4E) [42]. The trajectories obtained closely resemble those obtained by means of the maximum flow algorithm. Interestingly, the gene states move “downhill” along the differentiation trajectories in the difference SOM, a picture, which resembles the epigenetic “Waddington” landscape [18,45]. This landscape was introduced as a conceptual model to illustrate differentiation of tissues from stem cells. Note that the SOM-landscape is a “real” expression data landscape, which this way supports the abstract Waddington concept of developing cells in gene-state space. In summary, our approach identifies cascades of gene modules changing expression upon differentiation in a tissue-specific (pseudo-)temporal order. In SOM metagene space it appears as “lava lamp”-like flows of upregulated genes, which can be summarized into gene expression trajectories directed from stemness toward differentiated tissue expression programs.

### 3.4. Development along Branched Trees

**Figure 5:**
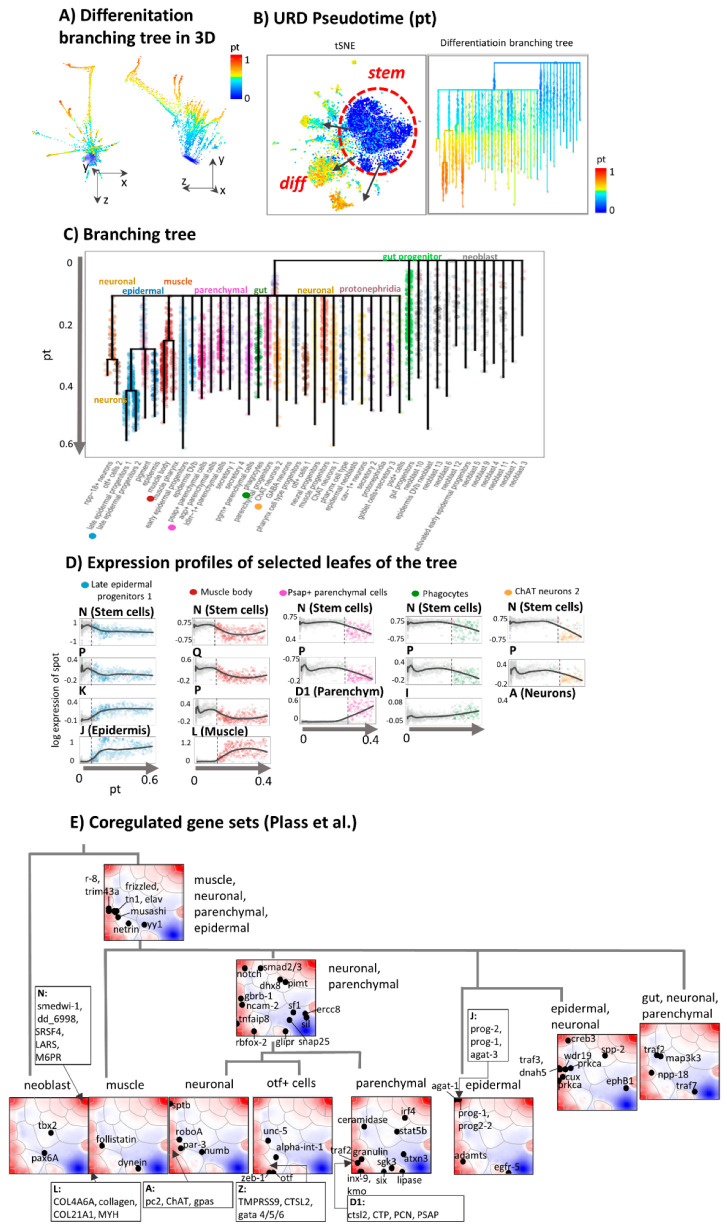
Branched pseudotime analysis of planarian tissue development. (**A**) URD plot [40] of branched differentiation in 3D is shown in two projections. The pseudotime is calculated as the average number of diffusion steps required to reach each cell from the root cell (neoblast 1) [40]. (**B**) Pseudotime coloring of t-SNE plot and URD-branching tree. In pt-units epidermal tissues are most distant from the root. (**C**) Branching tree of cell differentiation. (**D**) Expression profiles of selected lineages along the branches of the URD differentiation tree. Samples are sorted along pseudotime (gray arrow). The black LOESS (locally weighted scatterplot regression)-curves serve as guide for the eye. Step-wise transitions between stemness and tissue transcriptional programs are shown by vertical dashed lines. (**E**) A tree of tissue-related gene clusters together with marker genes were taken from [29]. Accordingly, 48 co-regulated gene sets assign to Planarian tissues and combinations of them. Most of these genes locate not in the spots referring to fully differentiated tissues. The top five genes in these clusters according to our analysis are listed in the figure (see boxes and also Table 1).

The unbranched pt approach applied in the previous subsection sorts the cells along one dimension independent of the cell type. A more realistic computational approach is provided by URD [40]. It splits the pt path of planarian tissue development into a tree-like, multibranched one. The algorithm applies diffusion-like random walks to search for graphs through cellular states. The obtained trajectory starts with a common trunk of neoblast cells for early pseudotime values at pt < 0.6 (blue color in Figure 5A,B). Subsequently it divides into tissue-specific branches at pt > 0.6 (yellow to red). Notably, the multibranched tree obtained by means of the URD algorithm reproduces the differentiated cell types identified by independent methods in the original publication [29]. Different visualizations of URD branching in terms of a 3D cell cloud (Figure 5A), of a path-tree or t-SNE plots (Figure 5B) enable inspection of paths under different perspectives. The tree describes differentiation into more than thirty final tissue types via 13 progenitor populations (Figure 5C). Initial stemness states stratify into a variety of neoblast and progenitor cells such as gut and early epidermal progenitors at pt = 0. Subsequently, the main branch splits the trajectory into sub-branches describing differentiation into tissues such as parenchymal, neuronal, proto-nephridial, and diverse epidermal and gut cells. Further branching separates late epidermal progenitors, pigment and epidermis cells, muscle body and muscle pharynx cells, and npp-18 and otf+ cell neurons.

The profiling of spot gene expression along the different sub-branches using URD-pseudotime (pt) as argument shows the decline of expression of the stem cell module “N” and the gain of the expression of the spot-modules referring to differentiated tissues as expected (Figure 5D). Differentiation proceeds rather step-wise which concerns variance of the data than continuously at different pt-values ranging from about pt = 0.1 (epidermal tissue) and 0.2 (muscle) to 0.5 (neurons) which suggests switching of the cellular machinery from stemness programs into programs of differentiated cells almost without intermediate expression states. Some tissues show indications of intermediate states (e.g., muscle, epidermis) in terms of more smooth transitions between undifferentiated source and differentiated sink states.

Next, we made use of previous stratification of planarian genes into 48 clusters of co-expressed genes, which were assigned to different tissues and tissue-combinations [29]. The tree in Figure 5E illustrates the obtained hierarchy and maps marker genes taken from [29] into the SOM. In general, one observes that genes upregulated in several tissues (upper part of the tree) locate more in the middle part of the SOM and of the intended trajectories while marker genes for single tissues are found in and near the respective tissue-related spots detected in our analysis. Overall, the spread of genes is relatively large, presumably because the number of 48 clusters used in [29] is insufficient to resolve the expression patterns underlying tissue differentiation. On the other hand, the location of part of the tissue markers slightly away from the sink nodes presumably reflects also the fact that they are activated in transition states as progenitors of more than one tissue type as visualized in the tree (Figure 5E). Tissue markers of our spot-analysis are listed in Figure 5E and Table 1. In summary, multi-branched pseudotime approaches such as URD provide an overview of tissue differentiation by sorting the cells into differentiation paths based on their mutual similarity relations.

### 3.5. RNA Velocity Trajectories

RNA-velocity analysis offers an independent approach of studying developmental dynamics. It calculates the change of mRNA abundance of each gene per unit of time in every single cell making use of the relation between spliced and unspliced mRNA and assumptions about the reaction kinetics of splicing [31]. RNA velocity is positive if the amount of mRNA increases. It refers to the situation when the amount of unspliced mRNA exceeds the amount of spliced mRNA compared with the steady state situation meaning that transcription and mRNA degradation are in equilibrium. Overall, one obtains a multidimensional RNA-velocity vector for each cell where each dimension refers to the rate change of transcription of one gene. The combination of velocities across genes then predicts the future expression state of the cell. In its original implication, the mRNA velocity vector points to direction of increasing mRNA abundance in sample space. For planarian single cell transcriptomics such plots show the expression state to which the cell is apparently moving in time [31].

Here we apply this concept also to expression space, where RNA velocity refers to the rate change of mRNA expression in each metagene-pixel of the SOM-space. It results in a vector whose components are given by rate changes of all individual genes contained in the metagene-cluster. The resulting RNA-velocity vector points in the direction of local mRNA rate changes in SOM space. Overall mRNA velocity analysis thus generates a metagene-based vector-field pointing toward increasing transcript abundance. It consequently points in the direction of “high” expression spot areas containing either genes upregulated in differentiated tissues such as epidermal (spot “J”), neuronal (“A”, “Y” and “F”), and muscular (“L”) tissues or genes upregulated in stem cells (module “N”). Hence, overexpression spots represent attractors of RNA-velocity (compare with Figure 3C). In turn, areas of invariant expression lack measurable RNA rate changes. Hence, RNA velocity analysis provides information about local slopes of mRNA abundance in gene expression space. Interestingly, RNA-velocity “vanishes” in the kernel-area of maximum expression of the spots meaning, that expression indeed reached its asymptotic maximum value.

**Figure 6:**
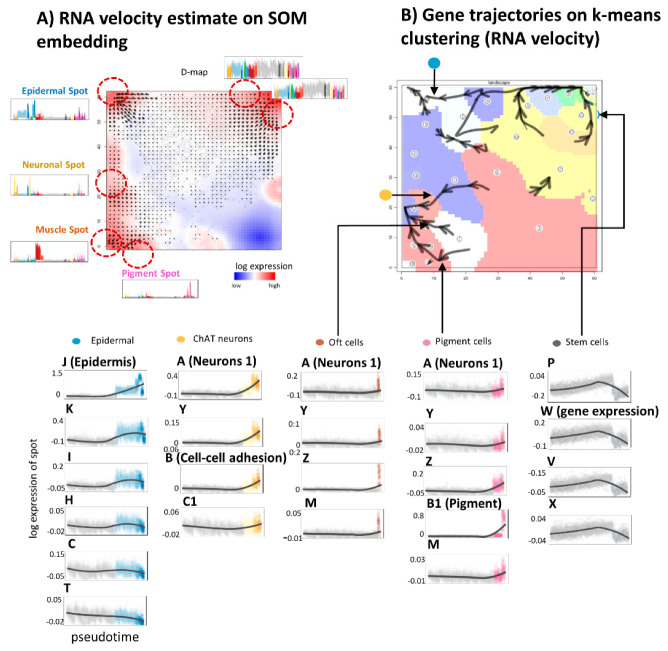
RNA velocity portrayal: (**A**) RNA velocity vector-field with metagene resolution identifies overexpression spot modules as attractors of overexpression except the “kernel” metagenes in the center of each spot (see text). (**B**) Velocity-trajectories using SOM area-segmentation indicate “watersheds” of differentiation between the attractors. The expression profiles illustrate expression changes along the selected trajectories.

In the next step, these vector field properties were transformed into “vector”-trajectories using the R program velocito [31]. It summarizes vectors along the main directions pointing toward the major attractor-modules separated by “watersheds” (Figure 6B). The trajectories obtained resemble the gene-state trajectories connecting source and sink modules. However, RNA-velocity trajectories are more diverse, particularly, showing branched paths which point toward the local attractors of overexpression. Taken together, both SOM-based dynamic and RNA velocity analysis provide similar trajectories indicating that differentiation points toward overexpression modules of differentiated tissue.

## 4. Discussion

Single-cell RNA-omics opens novel opportunities for molecular profiling of thousands of individual cells in parallel. The analysis of cellular heterogeneity and of patterns across such molecular landscapes still faces challenges. Current computational approaches mostly share the following workflow components: (1) dimensionality reduction to extract most relevant axes of variability, (2) cluster and diversity analysis to extract most relevant groups of cells and similarity relations between them, and (3) feature selection to extract most relevant genes as markers characterizing the different cell types. Assumptions about temporal development enabled to perform diversity analysis of cross sectional data in such a way that one can deduce information about the (temporal) dynamics of cellular development and adaptation to changing environmental conditions. State-of-the-art methods accomplish this task in cell-state space, i.e., in the multidimensional manifold spanned by all possible states that cells can adopt upon development and changing environmental conditions. Hereby, i.e., by using scRNAseq data, the particular “state of the cell” is given by its transcriptional phenotype, i.e., the entirety of transcripts produced by a cell in a certain developmental and environmental situation and measured by the scRNAseq technology applied. We here “transpose” this view from the state space of the cells into the space of transcriptional patterns, which are derived from the scRNAseq data without linking gene expression directly to cell states.

From a methodical perspective, we complemented our SOM portrayal providing static transcriptomic landscapes by this dynamic aspect in order to deduce (pseudo-)temporal information from snapshot data obtained in cross sectional studies. Using SOM-portrayal we identified tissue-specific clusters of activated genes and linear trajectories linking the stemness-source and tissue-related sink clusters expressing transcriptional programs, which reflect the specific functional requirements of the respective tissue types. In addition, part of the sink spot-patterns reveal common activation of cellular functions in different tissues in support of our initial statement that cell- and gene-states are closely related but do not match in an one-to-one fashion. For example, morphogenesis-related genes (spot F) were activated in neuronal and muscle tissues as well, meaning that one and the same transcriptional program can contribute to different cell states. Our approach explicitly supports identification of such common gene states. Overall, the gene-state landscape decomposes into a directed graph describing development via linear and partly via branched paths. RNA velocity constitutes an independent approach to forecast future expression states. In the SOM landscape RNA velocity vectors point in the direction of the local slope of gene expression. This result seems reasonable and confirms this approach as an independent calculus for transcriptomic trajectories where the generated vector field provides the detailed shape of the local attractor fields pointing toward the overexpression spots.

We have chosen the Planarian example because it provides a multibranched lineage tree of tissue differentiation and also because for the first time it demonstrated the impact of single cell transcriptomics for establishing such lineage trees in a multicell organism [29]. Meanwhile a series of whole-organism and organ systems datasets have been generated, e.g., for Nematodes, Sea Anemones, Hydra, and Annelids, as well as the human hematopoietic system, lung, kidney, heart, gut endoderm, mesoderm, nervous system, and neural crest mostly including embryonic and adult tissue stadiums (see [16] and references cited therein). These transcriptomes contain information about multiple aspects of cell identity as provided by activated transcriptional programs (for example, cell cycle phases, metabolic states, cell-specific and tissue-specific molecular signatures). Hence, gene-state space-based “functional” views using SOM trajectories are expected to complement developmental paths in cell-state space in all these applications.

Novel developments further extend single cell technologies toward capturing other molecular features such as chromatin accessibility [46], DNA-methylomes, proteomes [47], and metabolomes [48], as well as multimodal measurements of them from the same single cells (e.g., mRNA and DNA [49]) and also in situ approaches complementing cell-intrinsic states with information on cell’s local environment [50]. Hence, from an integrative perspective these diverse features reinforce an extended view of cell states as multidimensional vectors with diverse pheno- and genotypic features as components, which will enable holistic and comprehensive analysis of developmental trajectories in feature state space. Conceptually such a multidimensional continuous “landscape” was inspired by Waddington’s epigenetic landscape [45]. Our gene-state space landscape can be interpreted as a gene-centric version of the Waddington-landscapes, which illustrates the development of cellular programs instead of cell types. Especially our difference construct between source and sink states generates SOM maps, which resemble the “down-hill along valleys” shape of the metaphoric epigenetic landscape in cell-state space.

We expect tumor progression as an important potential application of developmental trajectories where in addition to cell types (e.g., malignant cells, T cells, cancer associated fibroblasts) further diversity is expected to reflect distinct tumor stages and/or clonal lineages [51,52,53]. States in gene expression space are more dynamic (e.g., phases of cell cycle or adaptation of cells to different environment) than cell types. Tumor biology is typically driven by such subpopulations of cells. Their transcriptomic states activate along developmental lineages related to drug resistance, metastasis, immune evasion, and immunotherapy [54]. The role of genes along the respective state-space trajectories can be split into environmental stimuli accomplishing either core or transient functions according to [55]. RNA velocity vector fields possibly can be interpreted in terms of such transient effects modulating the local shape of the trajectories.

## 5. Conclusions

We here developed a method to “transpose” the concept of developmental trajectories in phase space of the cells into their phenotype space spanned by phenotype variables such as the expression of ten thousands of genes. After our proof-of-principle study using RNAseq data describing tissue development in the flatworm as example we see applications in different fields of biology and medicine, to other than transcriptomics data and also further needs for refinements of the method, e.g., to better disentangle the topology of the feature landscape and to link both, cell- and gene-state space trajectories.

## Figures and Tables

**Table 1:** Overview of module-wise enriched gene sets and marker genes of planarian sc RNASeq data.

Cluster	Genes	Name	Enriched BP Gene Sets (log p Enrichment)	Marker Genes	Tissue
A	235	Neurons 1	Neurotransmitter secretion (−6) Synaptic vesicle localization (−5) Neurotransmitter transport (−5) Regulation of neurotransmitter levels (−5) Synaptic vesicle transport (−4)	pc2, ChAT, gpas	cav-1+ neurons, ChAT neurons 1, ChAT neurons 2
B	1314		Phosphorus metabolic process (−4) Phosphate-containing compound metabolic process (−4) Homopholic cell adhesion via plasma membrane adhesion molecules (−4) Cell-cell adhesion via plasma-membrane adhesion molecules (−4) Cell communication (−3)	GABRB3 (dd21541), CAVII-like, th	
C	206				
D	265	Pharynx		vim-1,VIT (dd1071), NPEPL1 (dd181)	Epidermis, Pharynx
E	383			1.G9.2, ifn	
F	370	Neurons 2	Microtubule-based movement (−18) Microtubule-based process (−15) cilium morphogenesis/organization/assembly (≥−14) movement of cell/subcellular component (−9)	pkd2l-2, rootletin (dd6573), cav-1	cav-1+ neurons, GABA neurons
G	218				
H	330			Post-2c	
I	228			nb.22.le	
J	111	Epidermis	actin filament capping (−2) negative regulation of actin filament depolymerization (−2)	prog-2, prog-1, agat-3	Epidermis, late epidermal progenitors 1/2
K	100			pds	
L	150	Muscle	muscle structure development (−9) tissue development (−8) actin filament-based process (−7) anatomical structure development (−6) muscle organ development (−6)	COL4A6A (dd2337), collagen, COL21A1 (dd9565)	Muscle body
M	1032			cali, if-1, HSPG2 (dd8356)	
N	121	Stem cells	translational elongation and termination (≥−79) cotranslational protein targeting to membrane (≥−73) protein targeting to ER (−72) Peptide and amid biosynthetic process (≥−71)	smedwi-1, dd_6998	Stem cells
O	143				
P	114			bruli	
Q	44			vasa-1	
R	119	Run specific	Unknown origin, presumably due to batch effects		Run specific cells
S	974			dd_5560, SAMD15 (dd19710), wntP-3	
T	404			sp-5	
U	235				
V	265			TYMS, gH4	
W	146		Gene expression (−8) Nucleic acid metabolic process (−7) Cellular nitrogen compound metabolic (−6) Cellular macromolecule metabolic process (−5) Nucleosome organization (−5)		
X	398			dd_13666	
Y	445			GLIPR1 (dd210), npp-18	
Z	575			TMPRSS9 (dd7966), CTSL2 (dd582), gata 4/5/6	
A1	18239		G−protein coupled receptor signaling pathway (≥−47) cell communication (−12) Signaling (−11) single organism signaling (−11)	glipr-1, PI16 (dd940), ASCL4 (dd1854)	
B1	181	Pigment	organonitrogen compound catabolic process (−6) tyrosine catabolic process (−5) small molecule metabolic process (−4) response to transition metal nanoparticle (−4) L-phenylalanine catabolic process (−4) erythrose 4−	pgbd-1, PSAPL1 (dd1706), KMO (dd7884)	Pigment cells
C1	458				
D1	262	Parenchym	regulation of intracellular signal transduction (−3) negative regulation of antigen receptor-mediated signaling pathway (−3)	ctsl2, CTP, PCN, dd_Smed_v6_3_0, PSAP	aqp+ parenchymal cells, pgrn+ parenchymal cells, psap+ parenchymal cells
E1	226	Phagocytes		mat	Phagocytes
F1	725	Goblet, secretory cells			Goblet and secretory cells

## Supplementary Materials

The following are available online at https://www.mdpi.com/2073-4425/11/10/1214/s1. Figure S1: Cellular markers and previous signatures of planarian single cell transcriptome, Figure S2: Positions of selected genes are shown in the map together with their expression profiles, Figure S3: Profiles and maps of tissue-wise differentially expressed genes and gene sets which were identified by [29], Table S1: References of selected marker genes of Figure S1, Table S2: Overview of module-wise enriched gene sets of the GO terms Biological Process, Cellular Component, Molecular function and Protein Domains.
