# Supplementary material for: Developmental scRNAseq Trajectories in Gene- and Cell-State Space—The Flatworm Example
Source: Genes (Basel). 2020 Oct 16;11(10):1214. doi: 10.3390/genes11101214 (PMC7603055; doi:10.3390/genes11101214)
Supplement: Supplementary file 1 [file genes-11-01214-s001.pdf]

Supplementary File 1

Developmental scRNAseq trajectories in gene and cell state space – the flatworm example

Maria Schmidt [1]*§, Henry Loeffler-Wirth [1], Hans Binder [1]*

[1] IZBI, Interdisciplinary Centre for Bioinformatics, Universität Leipzig, Härtelstr. 16 – 18, 04107 Leipzig, Germany

* Correspondence: binder@izbi.uni-leipzig.de (H.B.); schmidt@izbi.uni-leipzig.de (MS); wirth@izbi.uni-leipzig.de

Content

1. Supplementary Figures 2
2. Supplementary Tables 6
3. References 13

1. Supplementary Figures

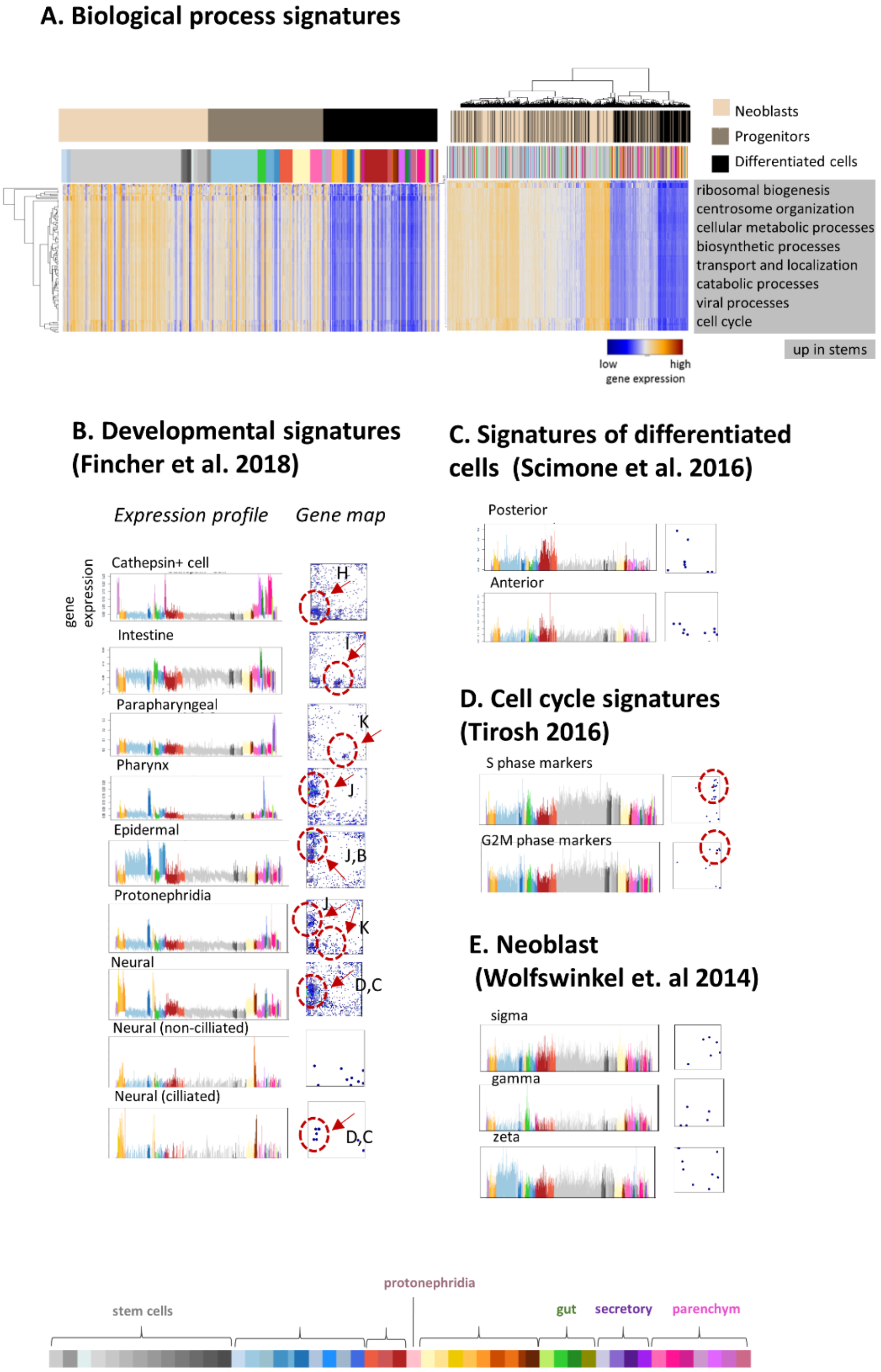

Figure S 1: Cellular markers and previous signatures of planarian single cell transcriptome: (A) Gene expression heatmap of signatures of the Gene Ontology (GO) - term biological processes related to processes like translation and biosynthesis show high expression in neoblasts as well as progenitor cells. GO information is taken from the planMine.de database . (B) Expression profiles and gene maps of cell type specific sets of genes taken from [1]. (C) Profiles and maps of several differentially expressed genes in posterior and anterior muscle cells that were identified by [2]. (D) Established cell cycle state markers from [3]. (E) Markers for previously characterized neoblast classes, σ-neoblasts (intestine progenitors), ζ-neoblasts (epidermal progenitors) and γ-neoblasts (nephridial, muscular, cns and eye progenitors) [4].

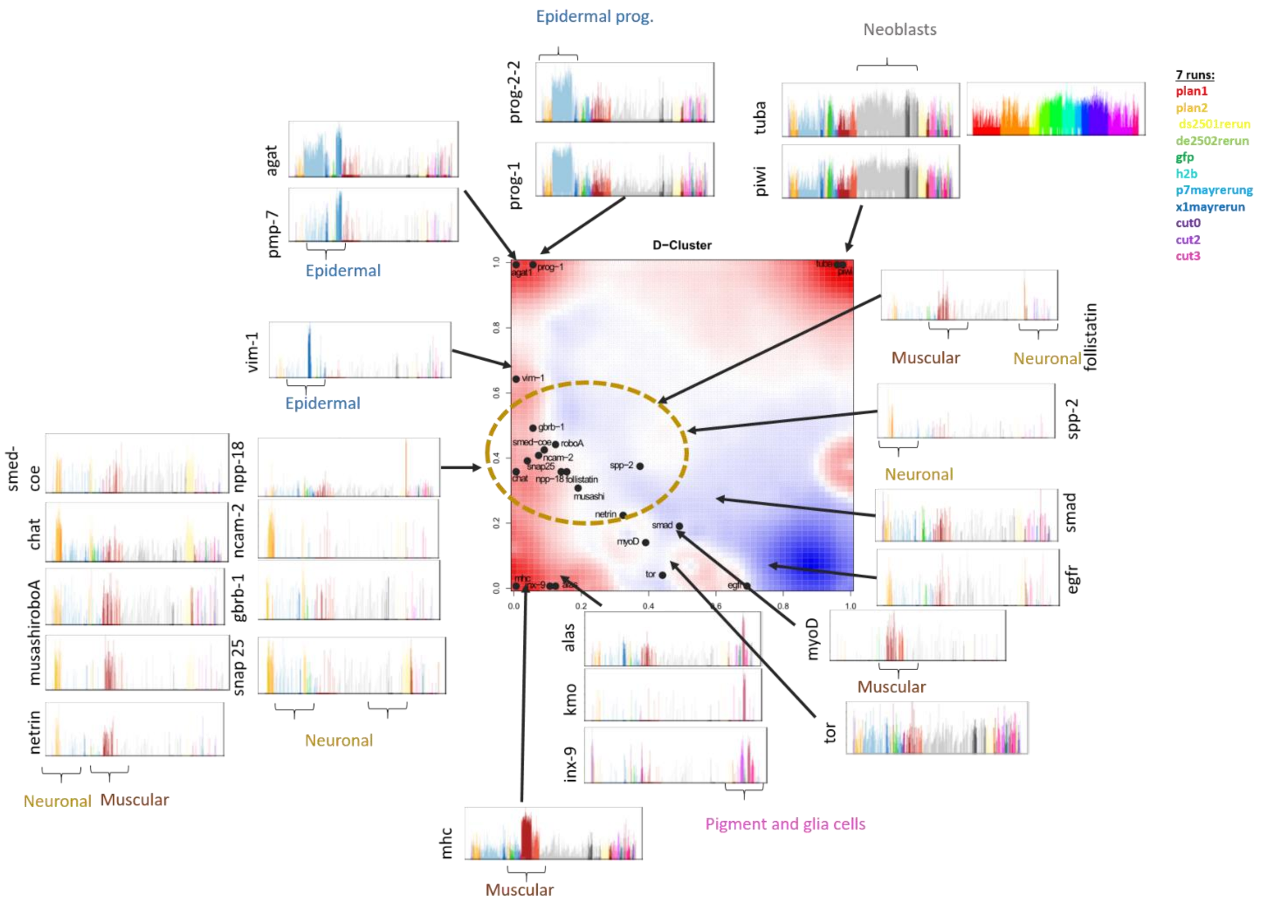

Figure S 2: Positions of selected genes are shown in the map together with their expression profiles (for References see **Table S 1**).

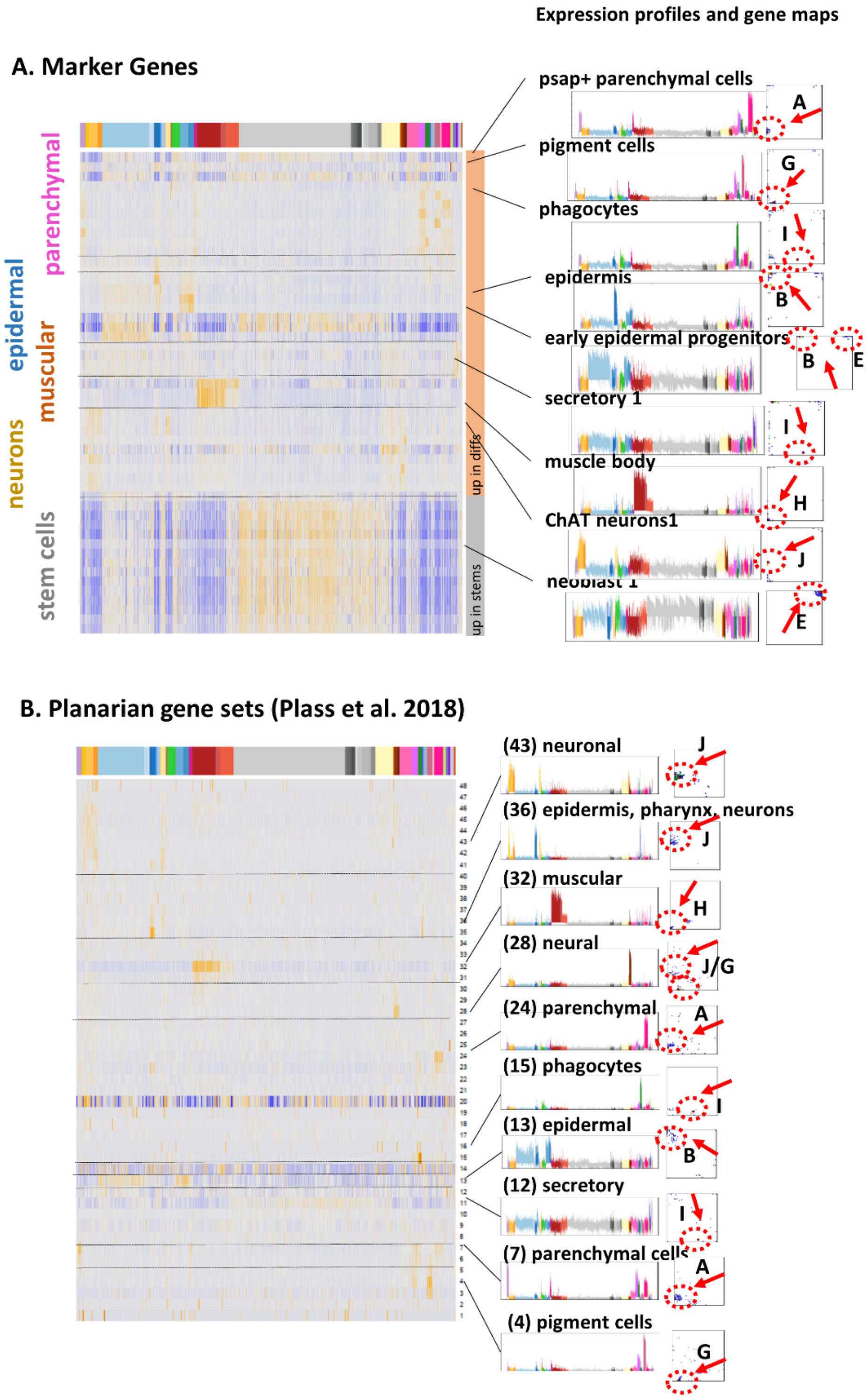

Figure S 3: Profiles and maps of tissue-wise differentially expressed genes and gene sets which were identified by [5].

2. Supplementary Tables

Table S 1: References of selected marker genes of **Figure S 1**.

ID	Name	pmid	Author	Journal	Year
dd_Smed_v6_1226_0	*alas*	27240733	Stubenhaus et al	Elife	2016
dd_Smed_v6_7884_0	*kmo*	27240733	Stubenhaus et al	Elife	2016
dd_Smed_v6_692_0	*inx-9*	16243308	Nogi and Levin	Dev Biol	2005
dd_Smed_v6_659_0	*piwi*	16311336	Reddien et al	Science	2005
dd_Smed_v6_648_0	*tuba*	22439894	Solana et al	Genome Biology	2012
dd_Smed_v6_332_0	*prog-1*	18786419	Eisenhoffer et al	Cell Stem Cell	2008
dd_Smed_v6_363_0	*prog-2-2*	26114597	Zhu et al	Elife	2015
dd_Smed_v6_920_0	*agat1*	18786419	Eisenhoffer et al	Cell Stem Cell	2008
dd_Smed_v6_1013_0	*pmp-7*	26114597	Zhu et al	Elife	2015
dd_Smed_v6_3921_0	*tor*	22479207	Gonzalez-Estevez et al	Plos Genetics	2012
dd_Smed_v6_1117_0	*npp-18*	20967238	Collins 3rd et al	Plos Biology	2010
dd_Smed_v6_6079_0	*smad*	23297191	Roberts-Galbraith et al	PNAS	2013
dd_Smed_v6_432_0	*mhc*	10079368	Cebria et al	Dev Biol	1999
dd_Smed_v6_12634_0	*myoD*	29168507	Scimone et al	Nature	2017
dd_Smed_v6_9584_0	*follistatin*	23297191	Roberts-Galbraith et al	PNAS	2013
dd_Smed_v6_11310_0	*egfr*	27325311	Barberan et al	Sci Rep	2016
dd_Smed_v6_364_0	*vim-1*	28292427	Wurtzel et al	Dev Cell	2017
dd_Smed_v6_10098_0	*spp-2*	20967238	Collins 3rd et al	Plos Biology	2010
dd_Smed_v6_8548_0	*ncam-2*	16629906	Fusaoka et al	Genes Cells	2006
dd_Smed_v6_19336_0	*gbrb-1*	25356635	Cowles et al	Plos Genetics	2014
dd_Smed_v6_6208_0	*chat*	24173799	Cowles et al	Development	2013
dd_Smed_v6_9893_0	*smed-coe*	25356635	Cowles et al	Plos Genetics	2014
dd_Smed_v6_3977_0	*snap25*	17547648	Takano et al	Dev Growth Differ	2007
dd_Smed_v6_8494_0	*roboA*	17251262	Cebria et al	Development	2007
dd_Smed_v6_14852_0	*netrin*	16033796	Cebria et al	Development	2005
dd_Smed_v6_13817_0	*musashi*	18440787	Higuchi et al	Mech Dev	2008

Table S 2: Overview of module-wise (M.) enriched gene sets of the GO terms Biological Process, Cellular Component, Molecular function and Protein Domains.

M.	Cells	Top GO term based gene set Biological process	Cellular Component	Molecular function	Protein Domain
**A**	Parenchym, Phagocytes		Lysosome (-9)<br>Lytic vacuole (-9)<br>Vacuole (-8)<br>Immunological synapse (-3)		Protein-tyrosine phosphatase, (-7)<br>Cystein peptidase (-6)
**B**	Epidermal cells		Spectrin (-4)<br>Fusome (-3)<br>cuticular plate (-3)<br>actin cytoskeleton (-3)		Dynein light chain, type ½ (-7)<br>EF-hand domain (-6)
**E**		translational elongation and termination (≥-79)<br>cotranslational protein targeting to membrane (≥-73)<br>protein targeting to ER (-72)<br>Peptide and amid biosynthetic process (≥-71)<br>nuclear-transcribed mRNA catabolic process, nonsense-mediated decay (-70)<br>peptide metabolic process (-67)<br>Viral transcription and gene expression (-65)<br>nuclear-transcribed mRNA catabolic process (-65)<br>cellular protein complex disassembly (-65)<br>multi-organism metabolic process (-65)	cytosolic ribosome (-96)<br>ribosomal subunit (-87)<br>cytosolic part (-84)<br>Ribosome (-81)<br>ribonucleoprotein complex (-63)<br>cytosolic large ribosomal subunit (-56)<br>large ribosomal subunit (-49)<br>cytosolic small ribosomal subunit (-33)<br>Cytosol (-28)<br>macromolecular complex (-23)<br>non-membrane-bounded organelle (-19)	structural constituent of ribosome (-84)<br>structural molecule activity (-55)<br>RNA binding (-30)<br>nucleic acid binding (-17)<br>heterocyclic compound binding (-5)<br>organic cyclic compound binding (-5)	Ribosomal protein L10/L12 (-5)<br>Translation protein SH3-like domain (-5)
**G**		organonitrogen compound catabolic process (-6)<br>tyrosine catabolic process (-5)			
**H**	Muscle	muscle structure development (-9)<br>tissue development (-8)<br>actin filament-based process (-7)	contractile fiber (-19)<br>Myofibril (-16)<br>Sarcomere (-16)<br>I band (-12)<br>Z disc (-10)<br>collagen trimer (-10)	structural constituent of muscle (-10)<br>extracellular matrix structural constituent (-8)	Collagen triple helixrepeat (-18)<br>Fibrillar collagen, C-terminal (-12)<br>Immunoglobulin subtype (-10)

	anatomical structure development (-6)<br>muscle organ development (-6)<br>cellular component morphogenesis (-6)<br>anatomical structure morphogenesis (-6)<br>actin cytoskeleton organization (-6)<br>organ development (-5)<br>developmental process (-5)	complex of collagen trimers (-8)<br>extracellular matrix component (-8)<br>actin cytoskeleton (-7)<br>extracellular matrix (-6)	structural molecule activity (-8)<br>cytoskeletal protein binding (-8)<br>actin binding (-8)	Collagen IV, non-collagenous (8)<br>Immunoglobulin-like fold (-7)<br>Immunoglobulin-like domain (-7)<br>Immunoglobulin subtype 2 (-6)<br>Immunoglobulin I-set (-5)
**A1 (k)**	G-protein coupled receptor signaling pathway (≥-47)<br>cell communication (-12)<br>Signaling (-11)<br>single organism signaling (-11)<br>signal transduction (-10)<br>ion transport (-9)<br>feeding behavior (-8)<br>Behavior (-7)<br>transmembrane transport (-7)<br>neurological system process (-7)	Integral/intrinsic component membran and plasma membrane (-≥33)<br>cell periphery (-12)<br>ion channel complex (-6)<br>transmembrane transporter complex (-6)	G-protein coupled receptor activity (-62)<br>transmembrane signaling receptor activity (-57)<br>signaling receptor activity (-54)<br>neurotransmitter receptor activity (-47)<br>signal transducer activity (-40)<br>molecular transducer activity (-39)<br>neuropeptide receptor activity (-36)<br>G-protein coupled peptide receptor activity (-34)<br>G-protein coupled amine receptor activity (-22)	GPCR, rhodopsin-like, 7TM (-158)<br>DDE superfamily endonuclease, CENP-B-like (-31)<br>Homeodomain-like (-29)<br>Ribonuclease H-like domain (-24)<br>HAT dimerisation domain, C-terminal (-17)<br>Homeobox domain (-15)<br>Tubulin (-12)<br>Na+ channel, amiloride-sensitive (-11)<br>Zinc finger, C2H2 (-7)<br>Innexin (-6)
**F (k)**	microtubule-based movement (-18)<br>microtubule-based process (-15)<br>cilium morphogenesis/organization/assembly (≥-14)<br>movement of cell/subcellular component (-9)<br>Cilium/flagellum-dependent cell motility (-7)<br>organelle assembly (-6)<br>Cell projection assembly,/organization/morphogenesis (-6)	Cilium (-26)<br>Cytoskeleton (-21)<br>cell projection (-14)<br>ciliary part (-13)<br>microtubule associated complex (-11)<br>Axoneme (-10)<br>ciliary cytoplasm (-10)<br>Microtubule (-10)<br>dynein complex (-10)<br>Centrosome (-5)	microtubule motor activity (-17)<br>motor activity (-15)<br>nucleoside-triphosphatase activity (-5)<br>pyrophosphatase activity (-5)<br>hydrolase activity, acting on acid anhydrides, in phosphorus-containing anhydrides (-5)	Dynein heavy chain (-12)<br>EF-hand domain (-6)

3. References

1. Fincher, C.T.; Wurtzel, O.; de Hoog, T.; Kravarik, K.M.; Reddien, P.W. Cell type transcriptome atlas for the planarian Schmidtea mediterranea. *Science (80-.).* **2018**, *360*.
2. Scimone, M.L.; Cote, L.E.; Rogers, T.; Reddien, P.W. Two FGFRL-Wnt circuits organize the planarian anteroposterior axis. *Elife* **2016**, *5*.
3. Tirosh, I.; Izar, B.; Prakadan, S.M.; Wadsworth, M.H.; Treacy, D.; Trombetta, J.J.; Rotem, A.; Rodman, C.; Lian, C.; Murphy, G.; et al. Dissecting the multicellular ecosystem of metastatic melanoma by single-cell RNA-seq. *Science (80-.).* **2016**, *352*, 189–196.
4. Van Wolfswinkel, J.C.; Wagner, D.E.; Reddien, P.W. Single-cell analysis reveals functionally distinct classes within the planarian stem cell compartment. *Cell Stem Cell* **2014**, *15*, 326–339.
5. Plass, M.; Solana, J.; Alexander Wolf, F.; Ayoub, S.; Misios, A.; Glažar, P.; Obermayer, B.; Theis, F.J.; Kocks, C.; Rajewsky, N. Cell type atlas and lineage tree of a whole complex animal by single-cell transcriptomics. *Science (80-.).* **2018**, *360*.
